# First case of superficial infection due *to Naganishia albida* (formerly *Cryptococcus albidus*) in Iran: A review of the literature

**DOI:** 10.29252/cmm.3.2.33

**Published:** 2017-06

**Authors:** S Aghaei Gharehbolagh, M Nasimi, S Agha Kuchak Afshari, Z Ghasemi, S Rezaie

**Affiliations:** 1 Department of Medical Mycology and Parasitology, School of Public Health, Tehran University of Medical Sciences, Tehran, Iran; 2 Department of Dermatology, Razi Hospital, Tehran University of Medical Sciences, Tehran, Iran; 3 Department of Medical Mycology, Razi Hospital, Tehran University of Medical Sciences, Tehran, Iran

**Keywords:** Cutaneous, *Cryptococcus albidus*, Infection, Itraconazole, *Naganishia albida*, Superficial

## Abstract

**Background and Purpose:**

*Naganishia albida* (formerly *Cryptococcus albidus*) is a non-neoformans cryptococcal species rarely isolated as a human pathogen.

**Case report:**

Herein, we present the case of a 26-year-old Iranian man with a superficial cutaneous lesion in the axilla. The initial treatment for pityriasis versicolor by clotrimazole was unsuccessful. We performed skin sampling based on the standard protocol and conducted further investigations by the conventional laboratory tests and molecular analysis of the skin samples. All the mentioned analyses revealed *N.* *albida* as the causative agent of infection. The minimum inhibitory concentration (MIC) analysis was carried out for the isolated agent, and the patient was treated using 100 mg daily of oral itraconazole.

**Conclusion:**

*N. albida *can be the causative agent of some superficial infections. This is the first report on the successful detection and treatment of a superficial skin infection due to *N. albida* by oral itraconazole.

## Introduction

*Cryptococcus* spp. are basidiomycetous yeasts considered as the responsible agents for a wide range of diseases, among which *C.* *neoformans* and *C. gattii* are mentioned as the most common pathogenic species [1-3]. However, the incidence of infection due to non-neoformans cryptococcal species such as *Papiliotrema laurentii *(formerly* C. laurentii*) and *Naganishia albida* has increased recently [4, 5]. *N. albida* is an encapsulated yeast occasionally detected on human skin, air, and soil [6, 7]. 

Some cases of infection caused by *N. albida* such as keratitis, pneumonia, encephalitis, and cutaneous and disseminated cryptococcosis have been reported [8-10]. The treatment regimen for systemic cryptococcosis is amphotericin B in combination with flucytosine followed by fluconazole as consolidation therapy [8, 11, 12]. To date, there is no defined treatment for superficial cryptococcal infection. In the current study, we presented the first case of superficial cutaneous infection caused by *N. albida*, as well as its successful treatment with 100 mg daily of oral itraconazole. To the best of our knowledge, this is the first report on superficial cutaneous infection due to *N. albida*.

## Case report

A 26-year-old Iranian man without any underlying diseases was referred to Razi Hospital, a referral center for skin diseases in Iran that is affiliated to Tehran University of Medical Sciences, with a hyperpigmented patch in the axilla. Based on clinical examination, the diagnosis was made as pityriasis versicolor. However, treatment with clotrimazole cream was not successful and he returned to the hospital after one month. 

**Figure 1:**
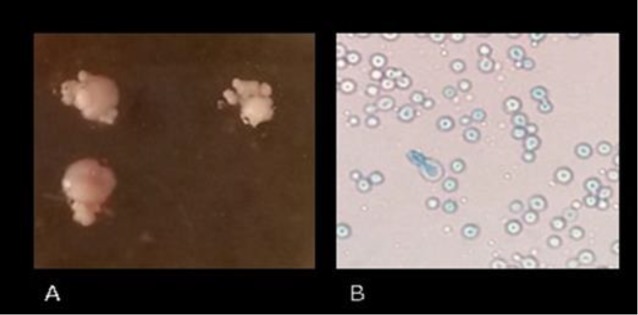
Macroscopic (A) and microscopic (B) demonstration of *N. albida*, which was indicated as the causative agent of superficial infection in the present case

Direct microscopic examination of the scales after mixing with 10% potassium hydroxide revealed the presence of yeast cells. Furthermore, the scales cultured on Dixon Agar (Quelab, Canada) plates for five days at 32°C produced white and creamy colonies with smooth surfaces. By Lactophenol Cotton Blue Staining of the smears, yeast cells similar to *Cryptococcus* were observed under microscope (Figure 1).

Genomic DNA was extracted from culture using glass beads method [13], and polymerase chain reaction (PCR) was performed using universal primers [14] of ITS1 (5'-TCC GTA GGT GAA CCT GCG G-3') and ITS4 (5'-TCC TCC GCT TAT TGA TAT GC-3') (Sinaclon, Iran). The PCR product with the approximate size of 600 bp was applied for sequencing (Macrogen, South Korea). Alignment of the obtained sequence in BLAST revealed high homology (99%) with *N. albida*, which is indicated with GenBank ID: MG020697.1.

In the following step, the minimum inhibitory concentration (MIC) values for itraconazole, voriconazole, and amphotericin B were determined using the microbroth dilution method according to the Clinical Laboratory Standards Institute (CLSI) standard protocols [15]. Briefly, the test was performed in 96-well round-bottom microtiter plates. Drug concentration ranges were 0.03 to 16 µg/ml for itraconazole, voriconazole, and amphotericin B. Yeast suspensions were prepared in RPMI-1640 medium and adjusted to provide a final inoculum concentration of about 0.5 × 10^3^ to 2.5 × 10^3^ cells/ml. The culture plates were then incubated at 35°C followed by reading after 48 h according to the M27-S3 supplement of the CLSI guideline [15]. The MIC results were then compared with a drug-free control culture plate. 

The MIC values for itraconazole, voriconazole, and amphotericin B were 0.062 µg/ml, 0.062 µg/ml, and 0.062 µg/ml, respectively, revealing the sensitivity of the mentioned causative agent. The patient was finally treated successfully using 100 mg daily of oral itraconazole. The Ethics Committee of Tehran University of Medical Sciences approved this report with the code No. IR.TUMS.SPH.REC.1396.2400.

## Discussion

*N. albida* is a non-neoformans species of the genus *Cryptococcus* with a similar morphology to *C. neoformans*. However, they can be differentiated by their reaction to biochemical tests such as phenol oxidase and color changes in Bird Seed Agar medium [9, 16-19]. In addition, *N. albida* is an opportunistic and encapsulated yeast found on human skin [6, 7]. Although *N. albida* rarely causes any diseases, there have been some case reports, including cases of meningitis, peritonitis, fungaemia, pulmonary and cutaneous infections, and keratitis (Table 1).

Moreover, cases of encephalitis, disseminated cryptococcosis, and pneumonia have been reported [4, 8, 20-29]. However, *N. albida* has never been reported as the causative agent of a superficial skin infection (Table 1). As can be observed in this table, the majority of reported cases were from the USA, whereas there was only one record from other countries such as Turkey. Besides, the most reported cases were isolated from blood (fungemia) and the most effective treatment protocol was with amphotericin B.

To the best of our knowledge, the case described here is the first report of a pityriasis versicolor-like superficial infection due to *N. albida*. In addition, treatment choices are limited for cryptococcal infections. The first choice of treatment for these infections is the combination of amphotericin B and flucytosine [11, 12]. Nonetheless, the treatment for infections due to *N. albida* is not well-defined, and amphotericin B has been mentioned to have limited efficacy in the treatment of *N. albida* [9]. *N. albida* shows various responses to different antifungal treatments. Therefore, there is no common treatment protocol for infections caused by this fungus.

**Table 1:** Overview of 24 reported cases of Naganishia albida (1972–2017)

**No**	**Age/sex/year**	**Location**	**Host status**	**Clinical presentation**	**Examination**	**Treatment**	**Outcome**	**Reference**
1	68/ M/ 1972	USA	Not indicated	Pulmonary	Culture	AmB	Cured	[20]
2	45/ M/ 1973	USA	Not indicated	Meningitis	India ink, Culture	AmB	Cured	[21]
3	29/M / 1980	USA	Juvenile rheumatoid arthritis, Alcoholic liver disease, receiving corticosteroids	Meningitis	Culture	AmB	Expired	[22]
4	65/ F/ 1987	USA	Acute myelogenous leukemia	Fungemia	India ink	AmB, 5FC	Expired	[23]
5	NA/NA/1989	NA	Pemphigus foliaceus, steroid therapy	Fungemia	Culture	KET	Cured	[24]
6	NA/NA/1993	USA	Receiving long-term hemodialysis	Renal disease	Culture	NA	NA	[25]
7	38/M / 1996	France	AIDS	Septicaemia	Culture	FLU, ITC	Expired	[26]
8	4/F / 1998	Tennessee	Acute Lymphocytic Leukemia	Fungemia	Not indicated	AmB	Cured	[27]
9	70/ M/ 2000	U.K.	Sézary syndrome, Noninsulin-dependent diabetes	Cutaneous	Culture, biopsy	FLU	Expired	[16]
10	73/ F/ 2004	USA	Rheumatoid arthritis	Pulmonary	Culture, biopsy	FLU	Not indicated	[1]
11	16/ F/ 2004	USA	AIDS	Scleral ulceration	Culture	AmB, ITC	Cured	[30]
12	23/M /2004	Korea	Renal transplant recipient	Disseminated	Biopsy, Culture	FLU	Cured	[17]
13	51/ M/ 2004	USA	Ddiabetes mellitus, lymphoma, Autologous progenitor cell transplant	Not indicated	Culture	AmB, ITC	Cured	[33]
14	69/F/2005	USA	Corneal transplantation	keratitis	Culture	Not indicated	Cured	[2]
15	44/ M/2007	Turkey	Acute respiratory failure	Pneumonia	Histopathology, Culture	AmB	Cured	[4]
16	14/M/2007	USA	Etanercept therapy	Cutaneous	Culture	FLU	Cured	[10]
17	NA/NA/2011	USA	Immunosuppressed, palmopustular psoriasis	NA	NA	NA	NA	[18]
18	0/ M/2011	Greece	Premature neonate	Fungemia	NA	AmB, 5FC	NA	[29]
19	55/M/2013	USA	liver transplant recipient	Fungemia	Culture	POS	Cured	[19]
20	28/M/2014	China	AIDS	Encephalitis	India ink	FLU	Expired	[9]
21	57/M/2015	USA	Peritoneal Dialysis, hepatitis C, type 2 diabetes	Peritonitis	Culture	AmB	Not indicated	[28]
22	45/M/2015	Taiwan	Hit by a plant	Keratitis	PCR	FLU, AmB	Cured	[8]
23	83/M/2017	Hungary	Receiving methylprednisolone	Cutaneous	Histopathology	FLU	Not indicated	[31]
24	26/M/2017	Iran	Immunocompetent	Superficial	Culture, PCR	ITC	Cured	Present case

M, male; F, female; AmB, amphotericin B; FLU, fluconazole; ITC, itraconazole; KET, ketoconazole; 5FC, 5 flucytosine; POS, posaconazole.

NA = not available (Original publication could not be accessed, information gathered from related abstracts)

Recently, it has been indicated that *N. albida* is only sensitive to amphotericin B and itraconazole [4, 30, 31]. Other reports revealed side effects due to the conventional amphotericin B (1 mg/kg/day) therapy against *N. albida*, which led the therapy to change to itraconazole [32]. Besides, successful treatment with fluconazole has been reported in patients with cutaneous infections caused by *N. albida *using a mean treatment period of 56 days [16].

 In the present case, after MIC approval, we chose 100 mg daily of itraconazole in a 10-day treatment period, which resulted in successful treatment. The three-month follow up of the patient revealed no relapse of the infection and no evidence of any clinical manifestations at the involved site. Furthermore, several factors such as the anatomical location of involvement, the power of immune system in host, and tissue damages can determine the proper medication and the required treatment duration for cryptococcal infections due to non-neoformans species [4, 5, 17, 33]. 

## Conclusion

In conclusion, *N. albida *was found to have the ability to cause superficial infections. The case presented here is the first report of successful detection and treatment of infection by this yeast. *N. albida *was detected by sequencing of ITS1-4 rDNA in this report and treated using oral itraconazole.
